# A Density-Dependent Target Stimulus for Inverse Bone (Re)modeling with Homogenized Finite Element Models

**DOI:** 10.1007/s10439-022-03104-x

**Published:** 2022-11-23

**Authors:** Sebastian Bachmann, Dieter H. Pahr, Alexander Synek

**Affiliations:** 1 Institute of Lightweight Design and Structural Biomechanics, TU Wien, Gumpendorfer Straße 7, 1060 Vienna, Austria; 2 Division Biomechanics, Karl Landsteiner University of Health Sciences, Dr. Karl-Dorrek-Straße 30, 3500 Krems, Austria

**Keywords:** Continuum, Distal radius, Trabecular bone, Load estimation, Physiological loads, Patient specific, Functional adaptation

## Abstract

Inverse bone (re)modeling (IBR) can infer physiological loading conditions from the bone microstructure. IBR scales unit loads, imposed on finite element (FE) models of a bone, such that the trabecular microstructure is homogeneously loaded and the difference to a target stimulus is minimized. Micro-FE (µFE) analyses are typically used to model the microstructure, but computationally more efficient, homogenized FE (hFE) models, where the microstructure is replaced by an equivalent continuum, could be used instead. However, also the target stimulus has to be translated from the tissue to the continuum level. In this study, a new continuum-level target stimulus relating relative bone density and strain energy density is proposed. It was applied using different types of hFE models to predict the physiological loading of 21 distal radii sections, which was subsequently compared to µFE-based IBR. The hFE models were able to correctly identify the dominant load direction and showed a high correlation of the predicted forces, but mean magnitude errors ranged from − 14.7 to 26.6% even for the best models. While µFE-based IBR can still be regarded as a gold standard, hFE-based IBR enables faster predictions, the usage of more sophisticated boundary conditions, and the usage of clinical images.

## Introduction

Finite element (FE) models of bone can be used for many applications, for example, to predict fracture risk or to optimize operative planning in orthopedic surgery.^39^ While it is relatively easy to create such models using generic geometry and boundary conditions, real bones are diverse in their shape, inner microstructure, and *in vivo* loading conditions. Hence, using patient-specific geometry, material, and boundary conditions is required to enhance the precision of those models.^37^ While there was a significant advancement in capturing patient-specific geometries and bone material models that account for microstructure, including physiological *in vivo* loading is still challenging. Physiological loading conditions can be measured *in vivo* using invasive technologies such as implanted strain gauges^33^ or instrumented endo-prostheses.^2,3^ While instrumented endo-protheses can measure the joint loading accurately, they are not used in healthy patients. Non-invasive options are musculoskeletal models^16^ which, however, require exact modeling of the muscles and bones of the patient.

Another approach to estimate physiological loading is to use the information stored in the microstructure of the bones. Bone undergoes constant repair and is also able to adapt to regular external loadings. Two mechanisms, known as remodeling and modeling, are responsible for changing the bone.^42^ While remodeling is determined by a coupled local bone resorption and formation, no such coupling can be observed during modeling. Often both modes cannot be discriminated, and the phenomenological response is then referred to as (re)modeling. Among other factors such as metabolism, it is driven by mechanosensitive cells^50^ inside the bone. Therefore, mechanical quantities can be measured inside the bone and used as a proxy for the (re)modeling response. One method that uses this relationship is inverse bone (re)modeling (IBR).^13,19^ Contrary to forward (re)modeling models, where the resulting microstructure is of interest when a particular load is applied, IBR can be used to find the loading that led to a given microstructure. Briefly, one possibility is to use FE models to impose a set of unit loads on the bone to measure the response in local mechanical quantities such as stress or strain energy density (SED). Then, the magnitude, direction, or superposition of these unit loads is varied until a nearly homogeneous loading state is reached, which is assumed to be close to a physiological loading in terms of the measured variable.

The first IBR algorithms were developed by Fischer *et al.*^19^ They used 2D FE models to predict joint loads and muscle forces at the proximal femur,^20–22^ to differentiate between coxa valga and vara,^18^ to predict relative bone loads at the distal radius and ulna,^17^ and to relate bone density to locomotor mode or activity.^6,7^ While Fischer *et al.* used relatively low-resolution quantitative-CT (~ 0.8 mm resolution) for measuring bone density, 2D homogenized FE (hFE) models and stress as the target stimulus, Christen *et al.*^13^ used micro-computed tomography (µCT) images, which depict the microstructure of bones in more detail, in 3D with SED as the target stimulus. Despite its simplicity, 3D IBR using µFE models was successfully applied to estimate physiological loading conditions for mouse vertebrae,^13^ mouse femora,^4^ human tibia,^10^ human vertebrae,^1^ predicting the reaction forces at the distal radius,^12^ and differentiate between species with different locomotor modes, using the hip^11^ or finger bones.^45^

While µFE-based IBR can be used to predict physiological loads on smaller bones (e.g., of mice or segments of bones), application to large bones (e.g., entire femur) is not a viable option due to high computational demands and resulting runtimes. Further, realistic boundary conditions, e.g., including articular contact, can only be modeled with high effort in µFE models^5^ but contact boundary conditions are readily available in most FE solvers when smooth meshes are used. Thus, only simplified boundary conditions are typically used in µFE, for example by using embedding materials^12^ or fully bonded articulated bones.^38^ Furthermore, due to the higher runtimes on large bones, also the number of load cases is limited in µFE-based IBR.^46^ Replacing the µFE models with hFE models would allow efficient IBR with realistic boundary conditions. However, so far, hFE-based IBR was limited to 2D models.^6,7,17–22^ In addition, no comparison between µFE- and hFE-based IBR has been performed so far.

The goal of this study was to translate the established IBR method for µFE by Christen *et al.*^13^ to hFE so that in the future physiological loading can also be estimated for larger bones with more realistic boundary conditions and lower computational demands. This study had two main objectives. First, to translate the tissue-level SED optimization to the continuum-level using a large set of trabecular bone cubes. Second, to test the new hFE-based IBR method on a set of distal radii sections and compare the hFE-based predictions of physiological loading to the gold standard, i.e., µFE-based predictions.

## Materials and Methods

### Outline

**Figure 1:**
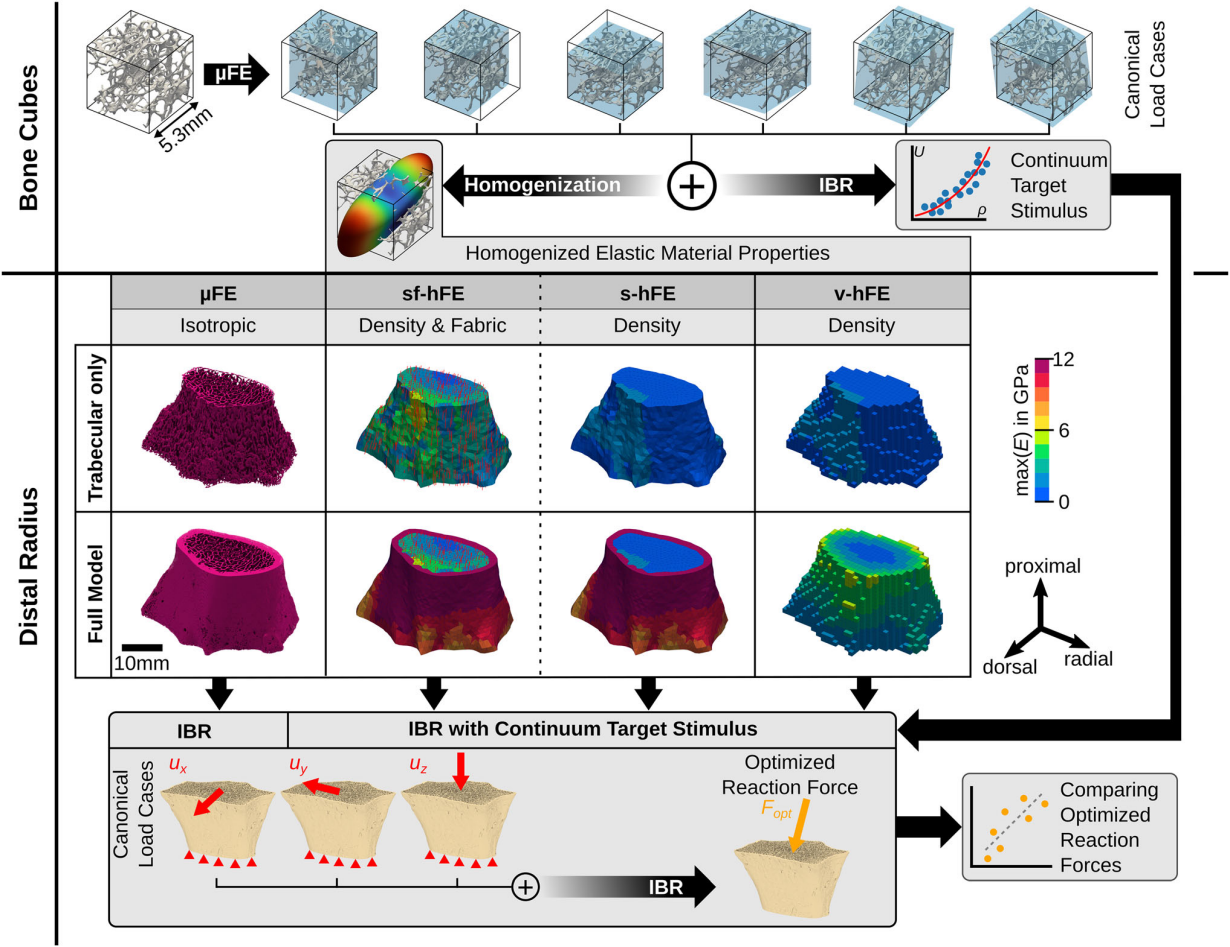
Graphical abstract of the study, which is split into two parts. In the first part, the continuum target stimulus is identified on trabecular bone cubes. This stimulus is required for the homogenized inverse bone remodeling (IBR) and applied in the second part, where µFE and hFE models are compared to each other in their ability to predict optimized reaction forces.

The study is separated into two major parts (Fig.  1). (1) A continuum target stimulus was identified on trabecular bone cubes ($n=701$) from various anatomical sites using kinematic uniform boundary conditions (KUBC) with six canonical load cases. These boundary conditions were also used in a previous study^28^ to identify homogenized elastic material properties. (2) 21 distal radius sections were modeled using four different types of finite element (FE) models: µFE models, smooth FE models with density and fabric dependent material mapping (sf-hFE), smooth FE models with only density-dependent material mapping (s-hFE), and voxel-based hFE (v-hFE) using density-dependent material mapping. For each of the four model types, versions with and without the cortex of the radius were created. Three canonical load cases using displacement boundary conditions were applied to each model, and inverse bone (re)modeling (IBR) was performed to predict physiological loading in terms of optimally scaled reaction forces. hFE models used the continuum target stimulus during the optimization.

### Theoretical Background

The original formulation of the µFE-based IBR by Christen *et al.*^13^ shall be repeated here briefly. A set of $n$ unit loads is applied to the bone. Then, unit load scaling factors ${\alpha }_{i}$ are found, such that the squared difference of local SED $U\left(x\right)$ and a tissue level target stimulus $\tilde{U}$ is minimized. These scaling factors are identified in an optimization procedure, using a residual function $r\left({s}_{i}\right)$ of SED scaling factors ${s}_{i}={\alpha }_{i}^{2}/n$. As SED is used, which cannot be negative, the optimization is subjected to only positive real numbers for ${s}_{i}$ and can be written as:

1 $${\mathop {{\text{min}}}\limits_{{s_{i} \in \mathbb{R}_{0}^{ + } }}} \;r\left( {s_{i} } \right) = \int {\left( {\sum\limits_{{i = 1}}^{n} {s_{i} } U_{i} \left( x \right) - \tilde{U}} \right)^{2} } dV$$

However, the tissue level target stimulus cannot be used for homogenized FE analysis, as elements are at the continuum level. Elastic constants of porous media can be related to the density using a power-law^9^ to form a relationship between tissue and continuum level. Therefore, the tissue level stimulus $\tilde{U}$ can be replaced by a continuum level stimulus ${\tilde{U}}_{\mathrm{hom}}$, which is a function of local relative density $\rho$: ${\tilde{U}}_{\mathrm{hom}}={\tilde{U}}_{0 }\rho {\left(x\right)}^{d}$. To ensure compatibility at $\rho =1$, ${\tilde{U}}_{0}$ is set to $\tilde{U}$. Inserting the power-law into Eq. (1) gives:

2 $${\mathop {{\text{min}}}\limits_{{s_{i} \in \mathbb{R}_{0}^{ + } }}} \;r\left( {s_{i} } \right) = \int {\left( {\sum\limits_{{i = 1}}^{n} {s_{i} } U_{i} \left( x \right) - \tilde{U}_{0} \rho \left( x \right)^{d} } \right)^{2} } dV$$

Note that the proposed continuum stimulus is therefore isotropic, although trabecular bone elasticity is orthotropic at the continuum level.^41^ Contrary to µFE, where all elements have the same volume, hFE meshes usually contain differently sized elements, and thus, the volume cannot be neglected in the equation. Equation (2) has to be rewritten in a discrete form to be used for FE and can further be transformed into a matrix equation to be directly solved by using a non-negative least squares solver.^30^ A detailed derivation is given in the Appendix.

The coefficient of variation (CV) is typically calculated before and after the optimization for the scaled SED ${U}_{\mathrm{scaled}}\left(x\right)={\sum }_{i=1}^{n}{s}_{i}{U}_{i}\left(x\right)$ to evaluate the effect of the optimization. Before the optimization, all scales were set to one, which gives the SED for unit scaled loads. The lower the CV, the higher the homogeneity of the load distribution.

### Bone Cubes: Parameter Identification for the Continuum Target Stimulus

The power-law relationship of density and continuum level target stimulus was identified on 701 µCT-images of bone cubes from a previous study.^28^ No new scans or experiments were conducted on the specimens for this study. The cubes were taken from various anatomical sites, had an edge length of 5.3 mm, and were scanned in a µCT with a resolution of 18 µm. The images were already segmented into bone and air voxels and directly converted to linear hexahedral elements. A linear elastic, isotropic bone material with $E=12\mathrm{GPa}$ and $\nu =0.3$ was used. Kinematic uniform boundary conditions (KUBC)^34^ were used with six canonical load cases (three in uniaxial compression and three in pure shear) with a normal and shear displacement of $-0.001\mathrm{mm}$.

The six canonical load cases were optimally scaled using the IBR (Eq. 1) with a tissue target stimulus of $\tilde{U}=0.02\mathrm{MPa}$, taken from literature.^13^ A non-negative least squares solver implemented in scipy^48^ (scipy.optimize.nnls) was used for the optimization. The optimized continuum stimulus $\langle {U}_{\mathrm{opt}}\rangle$ for each cube was calculated as $\langle {U}_{\mathrm{opt}}\rangle =0.5\sum_{i=1}^{6}{s}_{i}\langle {{\varvec{\sigma}}}_{i}\rangle :\langle {{\varvec{\varepsilon}}}_{i}\rangle$ using the volume averaged microscopic stress $\langle {{\varvec{\sigma}}}_{i}\rangle$ and *a-priori* strain $\langle {{\varvec{\varepsilon}}}_{i}\rangle$. Finally, the optimized continuum stimulus of each bone cube was plotted over the density $\rho$, and least-squares curve fitting was used to identify the exponent $d$ of the power-law relationship (see Eq. (2)).

### Distal Radius: Comparison of µFE and hFE-Based IBR

**Figure 2:**
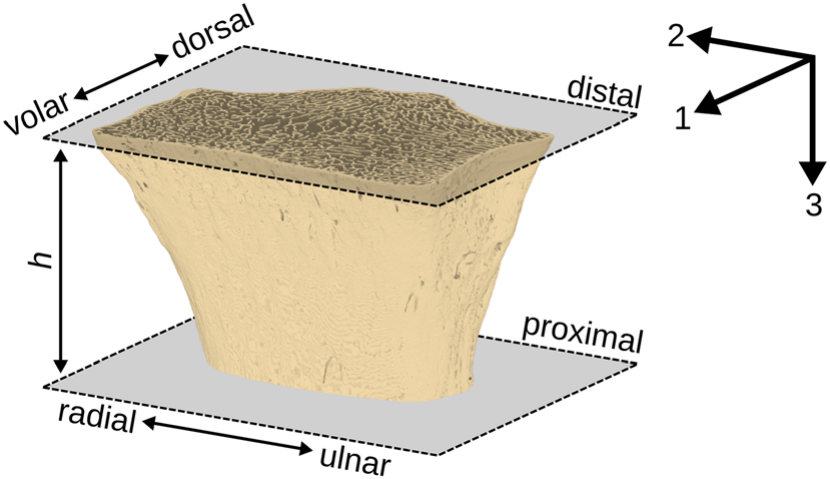
Alignment of the distal radii segments in the image frame. The average height $h$ of the radii was 19.22 mm. The bones are aligned such that the volar surface is parallel to the 2-axis of the image, and the axial direction is parallel to the 3-axis. Volar, radial, and proximal corresponds to the positive 1, 2, and 3-direction, respectively. The boundary conditions of the FE model are applied to the nodes of the proximal and distal faces of the bone, which are coplanar with the shown planes.

A µCT-image set of 21 distal radius sections from a previous study^29,44^ were used to test the new continuum level target stimulus. No new scans or experiments were conducted on the specimens for this study. The image resolution was 32.8 µm, with an average section height of 19.22 mm. The anatomical axes of the bones were aligned to the image coordinate system to retrieve comparable results (Fig.  2). The axial direction was already aligned during scanning to the 3-axis. The volar surface was manually aligned along the 2-axis.

Two sets of models were created to investigate if the cortex added any bias in the IBR. The first set contained only the trabecular bone without the cortex, while the second set included both trabecular bone as well as the cortex. As homogenized material properties were only identified on trabecular bone samples, the goal of this reduced model was to find out if the homogenization of trabecular bone yielded the same results as µFE, without the influence of the dense cortex.

The µFE models were created similar to the bone cubes, using a direct voxel to linear hexahedral element conversion and a linear, isotropic material with $E=12\mathrm{GPa}$ and $\nu =0.3$. Two model types were created: one including the cortex (full model) and one without the cortex (trabecular-only). Three load cases (compression in 3-direction, shear in 23 and 13 plane) were applied by imposing a displacement of 0.01 mm magnitude on the nodes of the distal plane in the respective direction. All nodes at the proximal plane were fixed in all three directions. The reaction force ${{\varvec{F}}}_{i}$ at the distal plane was calculated for each load case.

Three different homogenized FE models were created from the radii sections using two different meshing methods and two different material mappings (see Fig.  1). Likewise, as in µFE, each model was created with and without the cortex. Smooth FE models^35^ (s-hFE and sf-hFE) were created similar to a previous study^47^ using quadratic tetrahedral elements for the trabecular bone and quadratic wedge elements for the cortical bone. Both had an element edge length of around 1 mm. Homogenized voxel FE models (v-hFE) were created similar to a previous study,^29^ using a regular grid of quadratic hexahedral elements with an element edge length of around 1 mm. The same boundary conditions as for the µFE models were applied.

An hFE material mapping algorithm^36^ (Sampling sphere diameter 5 mm, background grid distance 2.5 mm) was used to map either a power-law-based density-dependent material or a Zysset-Curnier type^51^ material, which is dependent on local fabric and density. Details of these models are presented in the Appendix. Elastic material constants for trabecular bone were already identified in a previous study^28^ using the same bone cubes as for the identification of the continuum target stimulus. The used base material constants are given in Table 1. Trabecular bone material properties of the smooth hFE models were mapped using local density only (s-hFE) or using density and fabric (sf-hFE). Cortical bone in both smooth models was modeled using a power-law-based density-dependent material. In the voxel hFE models (v-hFE), material properties were mapped using only density for both trabecular and cortical bone.

**Table 1:** Elastic base material constants used for the material mapping.

Type	${E}_{0}$ in MPa	${\mu }_{0}$ in MPa	${\nu }_{0}$	$k$	$l$
Trabecular Bone (Density + Fabric)	10,320.4	3470.7	0.2278	1.62	1.1
Trabecular Bone (Density)	8812.8	3536.0	–	1.63	–
Cortical Bone (Density)	12,000.0	4615.4	–	1.63	–

The constants are used in the respective material model type to determine the local material properties from density and/or fabric as presented in the appendix

Inverse bone (re)modeling, including the previously identified exponent $d$ (Eq. (2)) for the continuum target stimulus, was applied to all radius sections and all four model types with and without cortex. Again a non-negative least squares solver (scipy.optimize.nnls) was used for the optimization. The resulting optimal scaling factors ${\alpha }_{i}$ were used to scale the three reaction forces ${{\varvec{F}}}_{i}$. First, unit scaled force ${{\varvec{F}}}_{\mathrm{unit}}=\sum_{i=1}^{3}{{\varvec{F}}}_{i}$ were calculated and, second, the optimized reaction forces were calculated as ${{\varvec{F}}}_{\mathrm{opt}}=\sum_{i=1}^{3}{\alpha }_{i}{{\varvec{F}}}_{i}$ to allow for a comparison of reaction forces before and after optimization.

The hFE models were then compared to the µFE models by linear regression of ${{\varvec{F}}}_{\mathrm{unit}}$ and ${{\varvec{F}}}_{\mathrm{opt}}$ respectively. The coefficient of determination and Lin’s concordance correlation coefficient (CCC)^31^ were calculated for each regression. Further, the magnitude of the optimized force was evaluated as well as the off-axis angle $\theta$ from the 3-axis, calculated as:

3 $$\theta ={\mathrm{cos}}^{-1}\left({\left(0, 0, 1\right)}^{T}\cdot {\widehat{{\varvec{F}}}}_{\mathrm{opt}}\right)$$

This angle gives a measure of the ratio between the magnitude of axial loading and shear loading, irrespective of the components. Two scores were defined to compare the similarity between µFE and hFE in terms of predicted force angle and magnitude. An angle score for the two vectors ${\varvec{a}}$ and ${\varvec{b}}$ is defined as:

4 $${S}_{a}\left({\varvec{a}}, {\varvec{b}}\right)=1-\frac{{\mathrm{cos}}^{-1}\left(\widehat{{\varvec{a}}}\cdot \widehat{{\varvec{b}}}\right)}{\pi }$$

Here, $\widehat{{\varvec{a}}}={\varvec{a}}/\Vert {\varvec{a}}\Vert$ denotes the unit vector of ${\varvec{a}}$. A magnitude score is defined as:

5 $${S}_{m}\left({\varvec{a}}, {\varvec{b}}\right)=1-\sqrt{\frac{{\left(\Vert {\varvec{a}}\Vert -\Vert {\varvec{b}}\Vert \right)}^{2}}{{\Vert {\varvec{a}}\Vert }^{2}+{\Vert {\varvec{b}}\Vert }^{2}}}$$

Both scores are one if the two vectors are equal and zero if the two vectors are dissimilar.

### Soft- and Hardware

All statistical analyses were performed using scipy^48^ 1.7.2 and Python 3.7.4 (Python Software Foundation, https://www.python.org). The hFE meshing and the material mapping were performed using medtool 4.5 (Dr. Pahr Ingenieurs e.U., Pfaffstätten, Austria, http://www.medtool.at). The µFE models were solved in ParOSol,^23^ and all hFE models were solved in Abaqus 2022 (Dassault Systèmes, Vélizy-Villacoublay, France). All analyses were performed on a dual AMD EPYC 7452 system.

## Results

### Bone Cubes

**Figure 3:**
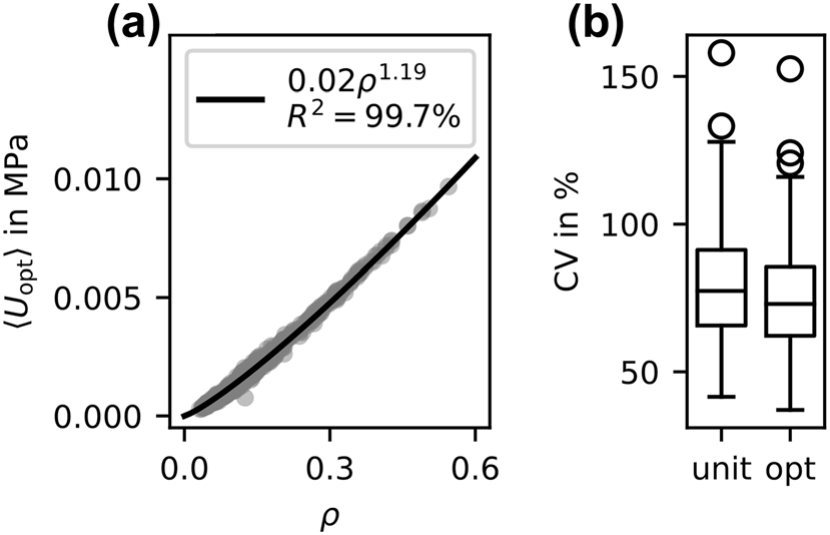
(a) Fitted power-law on the optimized continuum stimulus for the bone cubes. (b) Coefficient of variation (CV) for the SED distribution of the bone cubes before (unit) and after optimization (opt).

The µFE-based IBR on the bone cubes could significantly reduce the coefficient of variation (CV) of the tissue level SED on average from 78.8 to 74.2% ($p<0.001$; Cohen’s d 0.263; Fig.  3b). The optimized continuum stimulus followed a power-law with an exponent of 1.19 and a high coefficient of determination (99.7%; Fig.  3a).

#### Distal Radius: Trabecular Bone Only

**Figure 4:**
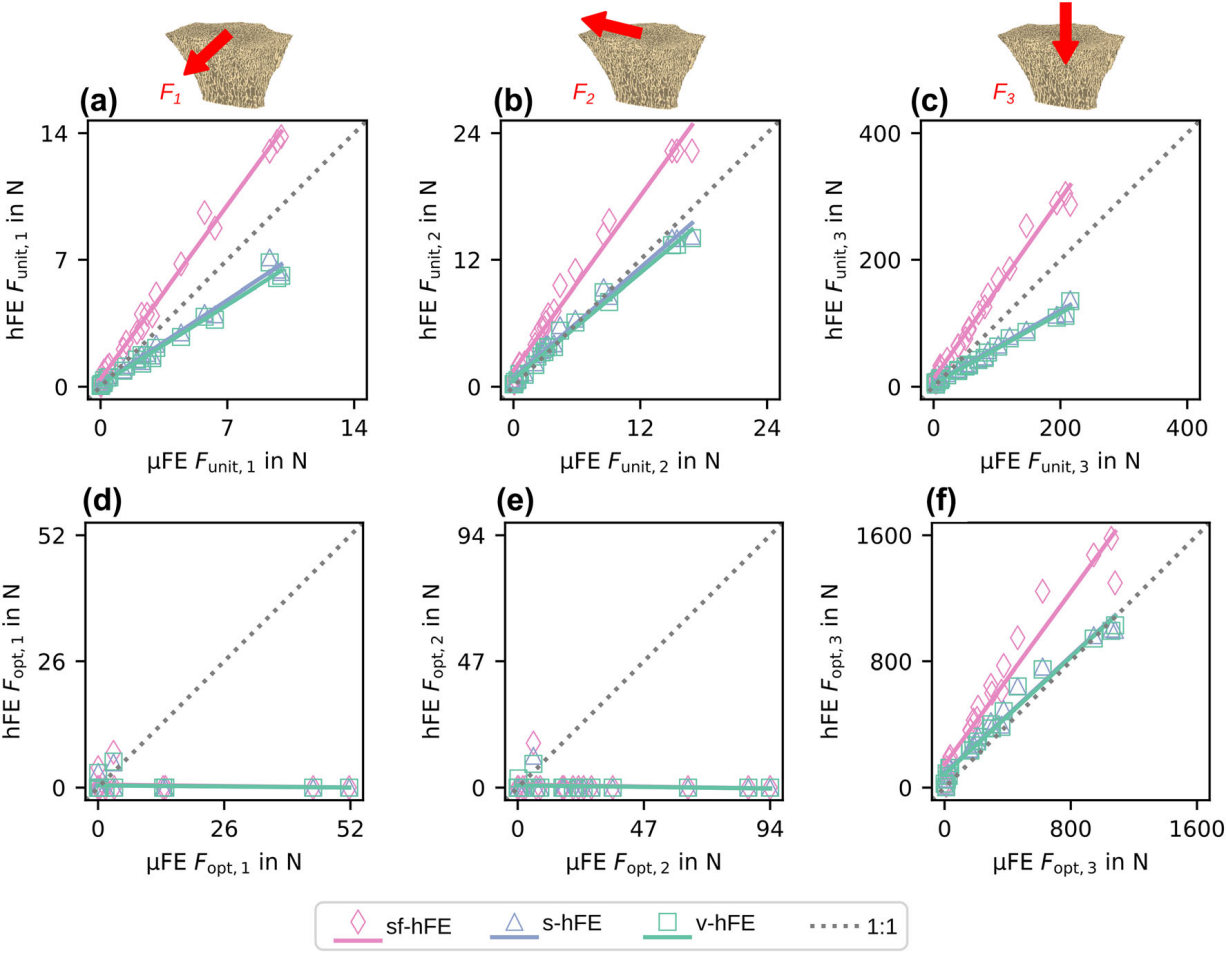
Trabecular-only model: (a-c) hFE over µFE unit scaled reaction force components. (d-f) Optimized reaction force components.

The predicted reaction forces ${\varvec{F}}_{\mathrm{unit}}$ before optimization (Figs.  4a–4c) differed between hFE and µFE for models of the radii without cortical shell. While both v-hFE and s-hFE underestimated the reaction forces, sf-hFE overestimated them. However, all hFE models had a good correlation of reaction forces with µFE, with R^2^ over 98% and good agreement in CCC with over 75% for all reaction force components (Table 2).

**Table 2:** Regression coefficients for trabecular-only model: Unit reaction forces.

		Slope	Intercept in N	*R*^2^ in %	CCC in %
sf-hFE	${F}_{\mathrm{unit},1}$	1.37	0.426	99.22	87.61
	${F}_{\mathrm{unit},2}$	1.38	1.53	97.91	82.46
	${F}_{\mathrm{unit},3}$	1.42	12.7	98.61	82.16
s-hFE	${F}_{\mathrm{unit},1}$	0.661	0.154	98.65	87.03
	${F}_{\mathrm{unit},2}$	0.864	0.872	98.45	98.17
	${F}_{\mathrm{unit},3}$	0.574	5.63	99.06	77.72
v-hFE	${F}_{\mathrm{unit},1}$	0.629	0.115	98.40	83.71
	${F}_{\mathrm{unit},2}$	0.837	0.708	98.83	97.96
	${F}_{\mathrm{unit},3}$	0.558	4.54	99.06	75.23

**Figure 5:**
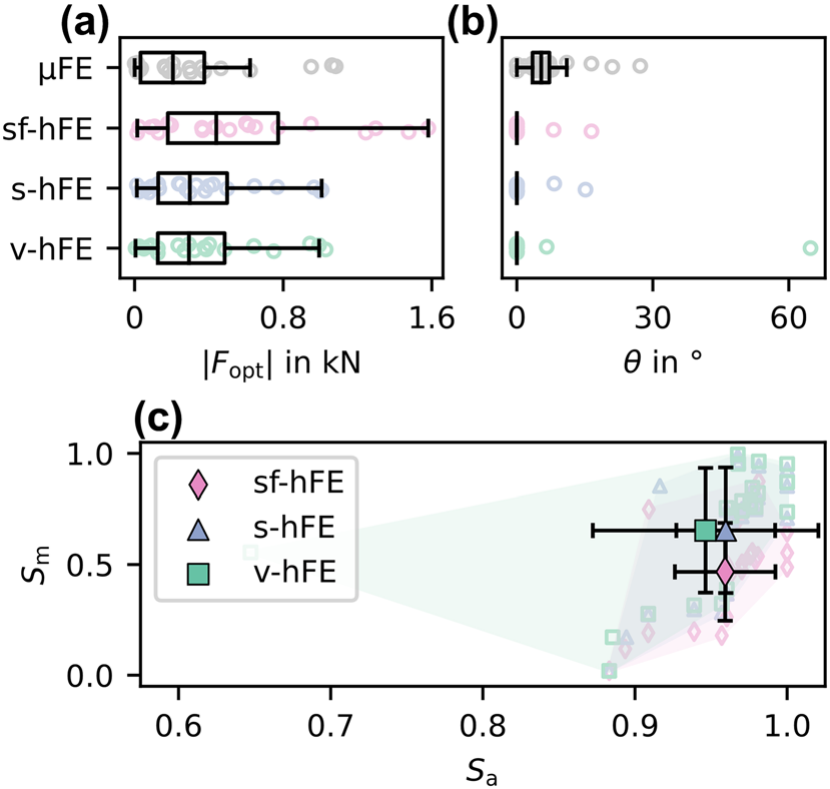
Trabecular-only model: (a) Optimized force magnitude and (b) angular difference towards the 3-axis for (c) angle (${S}_{a}$) and magnitude similarity score (${S}_{m}$). The marker gives the mean value, with the standard deviation as error bars.

After optimization (Figs.  4d–4f), µFE-based IBR identified the force component in the 3-direction (normal force) as the dominant load direction. Forces in 3-direction were 312 N on average, while shear components were much smaller with 6.2 N (1-direction) and 20.7 N (2-direction) on average. As a result, the off-axis angle was small, with an average of 7° (Fig.  5b). hFE-based IBR was able to identify the dominant load direction, but shear forces did not agree well with µFE-based IBR. As shear forces could not be captured by the hFE models, only the correlation of the force in the 3-direction was high, with R^2^ over 94% and CCC over 75% (Table 3). Both smooth hFE models showed a better similarity in angle, while v-hFE and s-hFE had a higher similarity in magnitude than sf-hFE (Fig.  5c).

**Table 3:** Regression coefficients for trabecular-only model: Optimized reaction forces.

		Slope	Intercept in N	*R*^2^ in %	CCC in %
sf-hFE	${F}_{\mathrm{opt}, 1}$	− 0.0114	0.614	0.86	− 1.94
	${F}_{\mathrm{opt}, 2}$	− 0.0158	1.12	1.48	− 2.04
	${F}_{\mathrm{opt}, 3}$	1.37	146	94.02	76.12
s-hFE	${F}_{\mathrm{opt}, 1}$	− 0.00862	0.456	0.92	− 1.46
	${F}_{\mathrm{opt}, 2}$	− 0.0113	0.799	1.48	− 1.45
	${F}_{\mathrm{opt}, 3}$	0.924	92.3	96.87	96.08
v-hFE	${F}_{\mathrm{opt}, 1}$	− 0.00842	0.453	0.86	− 1.43
	${F}_{\mathrm{opt}, 2}$	− 0.0129	0.853	3.14	− 1.67
	${F}_{\mathrm{opt}, 3}$	0.926	84.8	97.26	96.68

The µFE models had 104 million degrees of freedom (DoF) on average and took 63.8 min to solve using 27 CPUs in parallel. The sf-hFE and s-hFE models had 89,800 DoF on average and took on average 46.8 s and 47.5 s to solve, respectively. The v-hFE models had 74,283 DoF and took 19.3 s to solve on average. All hFE models used 4 CPUs in parallel.

### Distal Radius: Full Models

**Figure 6:**
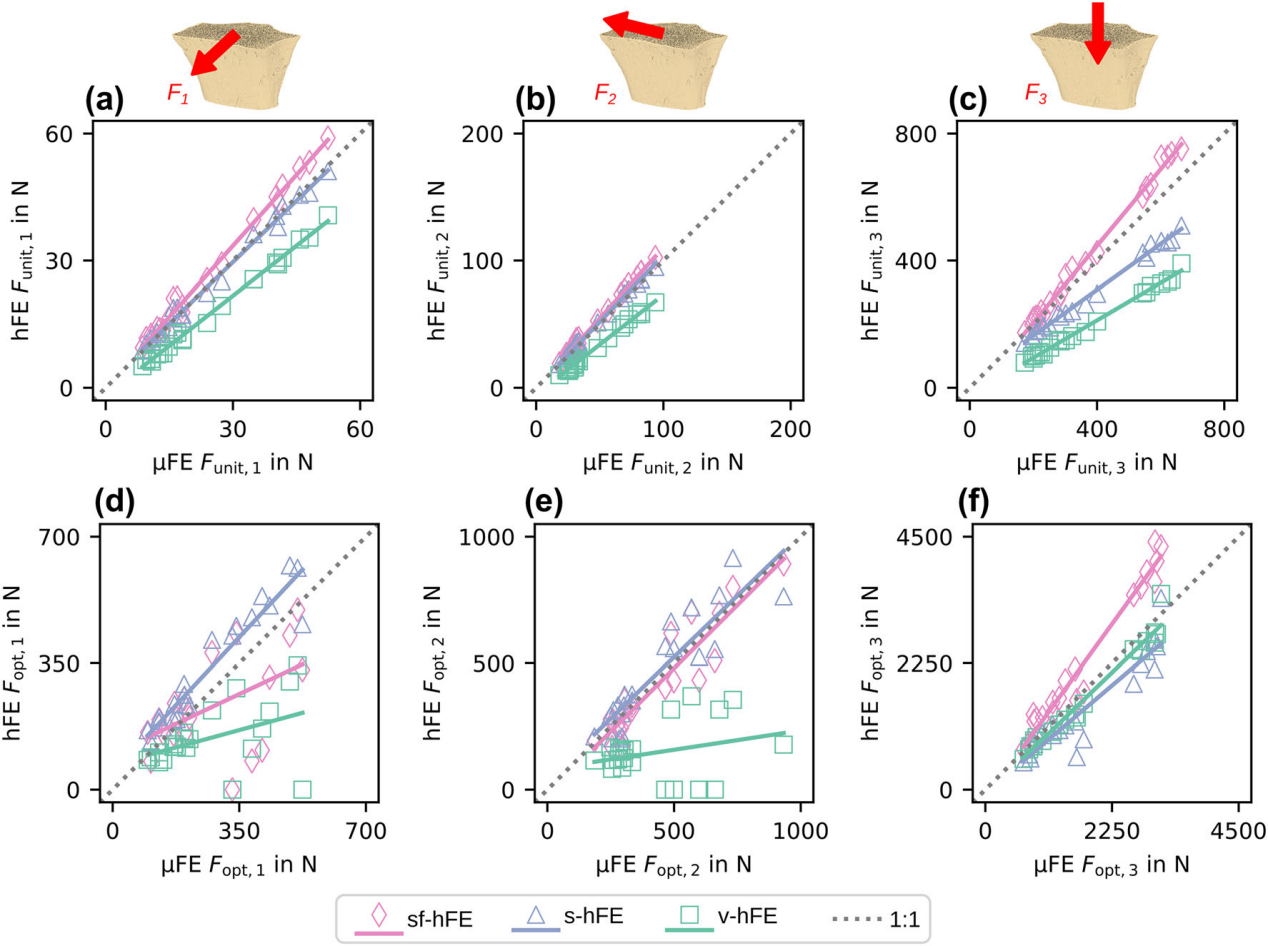
Full model: (a-c) Unit scaled reaction force components. (d-f) Optimized reaction force components for the full model.

Similar to the models without cortex, reaction forces before optimization (${\varvec{F}}_{\mathrm{unit}}$) correlated well between hFE and µFE models but showed over or underestimation (Figs.  6a–6c). While sf-hFE had higher unit reaction forces on average, both s-hFE and v-hFE underestimated the reaction force. All hFE models correlated well with µFE, with R^2^ over 98%. CCC was over 97% for both smooth hFE models, except for the 3-direction in s-hFE and all components for v-hFE (Table 4). Contrary to the radius without cortex, v-hFE and s-hFE showed different reaction forces.

**Table 4:** Regression coefficients for full model: Unit reaction forces.

		Slope	Intercept in N	*R*^*2*^ in %	CCC in %
sf-hFE	${F}_{\mathrm{unit},1}$	1.12	− 0.047	99.32	97.28
	${F}_{\mathrm{unit},2}$	1.11	− 1.08	99.73	97.93
	${F}_{\mathrm{unit},3}$	1.21	− 36.3	99.43	95.66
s-hFE	${F}_{\mathrm{unit},1}$	0.967	0.641	98.99	99.45
	${F}_{\mathrm{unit},2}$	1.04	− 0.175	99.64	99.46
	${F}_{\mathrm{unit},3}$	0.72	20.3	99.03	81.39
v-hFE	${F}_{\mathrm{unit},1}$	0.786	− 1.86	99.38	83.98
	${F}_{\mathrm{unit},2}$	0.785	−5.21	99.52	77.39
	${F}_{\mathrm{unit},3}$	0.588	−23.1	99.35	48.34

**Figure 7:**
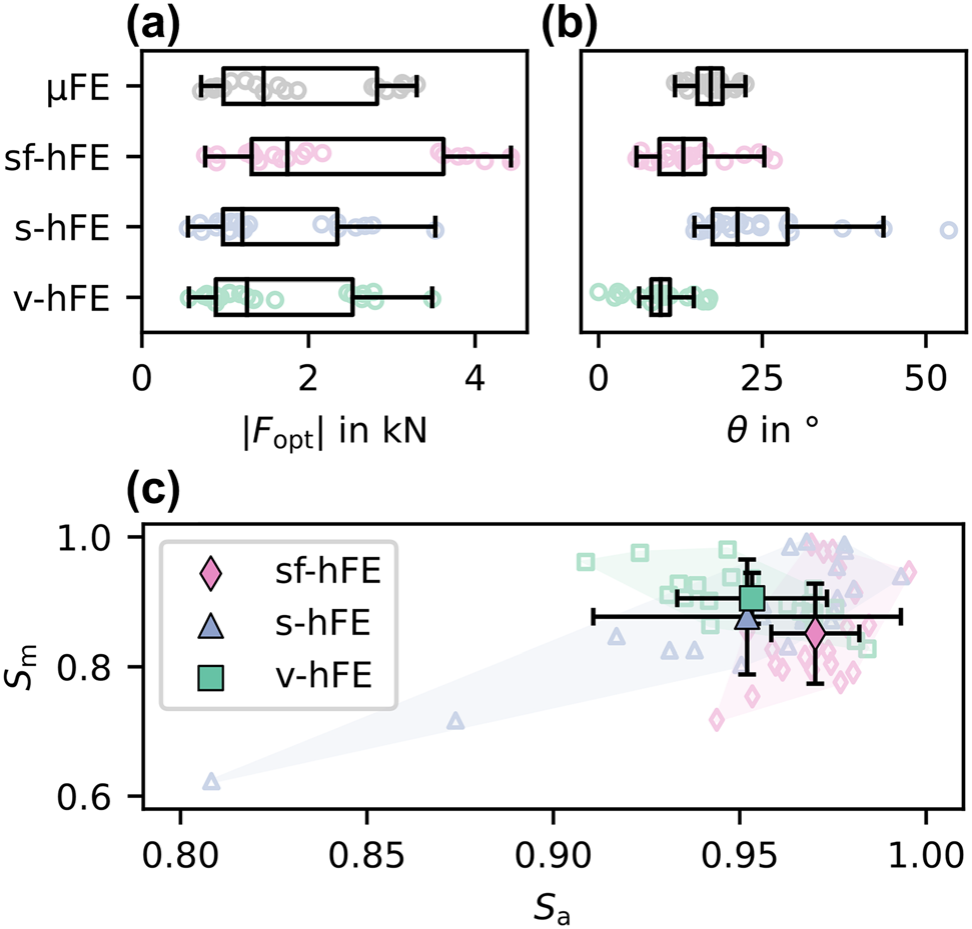
Full model: (a) Optimized reaction force magnitude and (b) angle towards the z-axis for the full model. (c) Angle (${S}_{a}$) and magnitude score (${S}_{m}$) for the full model. sf-hFE shows the smallest deviation and the highest similarity in angle, while s-hFE and v-hFE show better similarity in magnitude. The marker gives the mean value, with the standard deviation as error bars.

After optimization (Figs.  6d–6f), µFE-based IBR showed results in agreement with the radius without cortex, but the overall load magnitudes were higher. Forces in 3-direction were dominant with an average of 1735.8 N, and shear forces were one order of magnitude lower with averages of 268.2 N and 428.5 N for the 1- and 2-direction, respectively. The average off-axis angle was 17° (Fig.  7b). hFE-based IBR also identified the 3-direction as dominant. In contrast to the radius without cortex, almost all hFE models also predicted shear forces in agreement with µFE-based IBR. While the magnitude of the optimized reaction force showed a similar pattern as for the models without cortex, the angle of the optimized reaction force was best predicted by sf-hFE (Fig.  7c). All models showed a high correlation for the optimized force (R^2^ > 81%, CCC > 84%) except for the 1-direction component in sf-hFE and the 1 and 2-direction for v-hFE (Table 5).

**Table 5:** Regression coefficients for full model: Optimized Reaction forces.

		Slope	Intercept in N	*R*^*2*^ in %	CCC in %
sf-hFE	${F}_{\mathrm{opt}, 1}$	0.471	99.9	26.80	49.05
	${F}_{\mathrm{opt}, 2}$	0.999	− 19.5	86.54	92.34
	${F}_{\mathrm{opt}, 3}$	1.39	− 176	96.83	84.12
s-hFE	${F}_{\mathrm{opt}, 1}$	1.07	46.6	91.24	86.23
	${F}_{\mathrm{opt}, 2}$	0.97	38.8	81.32	89.20
	${F}_{\mathrm{opt}, 3}$	0.852	− 62.8	85.82	86.25
v-hFE	${F}_{\mathrm{opt}, 1}$	0.274	69.1	19.29	24.76
	${F}_{\mathrm{opt}, 2}$	0.152	81.8	7.38	8.97
	${F}_{\mathrm{opt}, 3}$	0.963	− 83.1	96.94	97.08

The µFE models had 198 million DoF on average and took 83 min to solve (on 27 CPUs), while sf-hFE and s-hFE had 107,624 DoF and took on average 67.8 s and 54.4 s to solve (on 4 CPUs). v-hFE had 88,386 DoF and took 24 s to solve on average.

## Discussion

This study presents a new method for homogenized inverse bone (re)modeling (IBR) on the basis of previously described µFE-based IBR to predict physiological *in vivo* loading for bones. The required relationship between the tissue level stimulus and the continuum level was found using a large sample of trabecular bone cubes with a high coefficient of determination. While all hFE models were able to predict the magnitude of the optimized reaction force with a good to high coefficient of determination, the angular accuracy varied between the different types of hFE models. Inclusion of the cortex had the highest influence on the model accuracy, while differences in material mapping or different meshing methods had less influence.

Although a good agreement between hFE and µFE-based load predictions was found for the distal radius, the used tissue stimulus function is still highly simplified as it only accounts for an accumulative and time-averaged stimulus of bone. While it showed the ability to predict physiological loading conditions using µFE methods on a variety of bones,^1,4,10–13,45,46^ other formulations can be used as well. Fischer *et al.*^19,20^ used continuum level effective stress, scaled to the tissue level, while the here employed method scales the stimulus to the continuum level and directly uses SED. Other possibilities to describe the stimulus function can be obtained by switching from a scalar to a higher-order quantity. For very small isotropic elements, as they are used in µFE, a more elaborate optimization criterion might be superfluous, whereas hFE might benefit from including more information as bone is known to be orthotropic and loaded in a multiaxial way at this scale.^41^ For instance, the optimal loading stimulus could include the orientation of principal stresses or the ratio of minimum and maximum principal stresses. Thus, future work could also test the viability of using different vectorial or tensorial quantities.

The here found exponent for the relation between tissue and continuum level was 1.19 and lower than used in the similar model of Fischer *et al.,*^19,20^ where an exponent of 2 was used. This relationship was found in experiments regarding bone strength^9^ and analytical models of porous structures.^27^ However, no comparison to other methods or validation of this assumption has been made so far. One explanation for this difference in value might be, that the exponent was fitted on a dataset, using the accumulated macroscopic SED as a target value, instead of resorting to proxy values such as bone strength. Thus, while bone strength might scale well with an exponent of 2, the accumulated SED might not. Further, the way the bone cubes are loaded also has an influence on the resulting SED distribution and thus also on the optimized values and finally on the accumulated SED.

An entirely different 3D hFE IBR approach was used by Campoli *et al.*^8^ and Garijo *et al.*^25^ They used forward remodeling models to train artificial neural networks (ANN) many different loading patterns that could then be used to predict the loading pattern in a given bone. While these models were able to work on larger bones, such as the proximal femur^8,25^ or the proximal tibia,^26^ they are only phenomenological, require highly time-consuming re-training for new load cases, and also individualized training for each bone. Also, the choice of the forward remodeling algorithm will influence the result, similar to the choice of the target stimulus in the here used model.

Using the computationally efficient (70 to 200 times faster than µFE) hFE-based IBR method presented in this study allowed the prediction of physiological loading of 21 distal radius samples with a high correlation to µFE based IBR if fabric and cortex is included in the model. While the correlation was high, the hFE models overestimated (sf-hFE) or underestimated (s-hFE, v-hFE) the unit reaction forces and subsequently also the optimally scaled reaction forces. This discrepancy was larger for the models that used only trabecular bone than it was for the full models, with the exception of v-hFE, which showed a higher deviation from µFE in the full model. There might be several reasons for these differences. First, the boundary conditions used to find the apparent stiffness can influence the homogenized elastic material properties. KUBC is known to overestimate the apparent stiffness.^15^ Other boundary condition types, such as PMUBC,^34^ could be tested instead. Second, the material mapping is based on trabecular bone cubes with a maximum relative density of 60%. Thus, for models that include elements with a higher density, the apparent properties might not match. This could be the case for v-hFE, where the cortex is averaged with the trabecular volume. Due to the high density-gradient between trabecular and cortical volume, smooth models (which model a sharp boundary between the volumes) work better in this respect. Different material mappings, which include a tissue function,^29^ could be applied in such cases. Last, micro-structural effects of low-density volumes can lead to a different result at the continuum level. Several radii had low-density regions (< 10% relative density) in the proximal region. Such effects are mitigated when the cortex is included.

The physiological reaction forces of the full distal radius section predicted by both hFE and µFE -based IBR were in a plausible region. The predicted off-axis force angle was similar to the one found by Smith *et al.*^43^ for the pushup load case *ex vivo*, but the predicted force magnitude was higher on average (1811 N for µFE with cortex) than estimated from that experiment (663 N). However, this magnitude is still in a physiological region estimated with up to 2410 N for power grip exercise.^40^ A few other studies also used IBR to predict distal radius loading. Walle *et al.*^49^ predicted physiological section forces using µFE-based IBR on a clinical µFE model of distal radius sections and found a similar pattern of optimized reaction force components, with smaller shear components (140 N and 280 N) than normal force (420 N). Conversely, Christen *et al.,*^12^ using the same algorithm, found results different from estimates in literature^40,43^ and the results of this study. Specifically, Christen *et al.* found high amounts of shear forces (45 to 465 N) and relatively low normal forces (1 to 235 N). However, different boundary conditions, by the addition of a soft connector layer, were applied to the models. Furthermore, also rotational load cases were added to predict moments, which was not the case in this study. Such load cases were not added in this study, as only µFE and hFE-based IBR were compared to each other and evaluating the moments is not strictly necessary.^14^

Differences between IBR-predicted and physiological load magnitudes could also be the result of the chosen target stimulus value. While the predicted load magnitude is influenced directly by the tissue target stimulus, the load angle (i.e., the ratio of force components) remains unaffected.^46^ In this study, a tissue target stimulus of 0.02 MPa was used.^13^ The value originates from the assumption that bone has to experience 2000µε to 3000µε of peak strain every day^32^ in order to maintain its mass, which can be converted to an SED when the material properties are known. For the herein used material properties, an effective strain for the current tissue stimulus is 1826µε and is thus in a realistic region for *in vivo* strains.^24^ Without additional information on the physiological range of strains, the tissue stimulus must be chosen arbitrarily or calibrated from *in vivo* data. For example, Christen *et al.*^10^ found a value of around 0.01 MPa (equivalent to 1715µε) for homeostasis at the distal tibia.

This study has some limitations. The hFE models were, so far, only tested on the distal radius sections. This location is characterized by relatively homogeneous trabecular bone predominately loaded in axial compression. In proximity to joints loaded in a multi-directional way, such as the proximal or distal femur, the hFE-based IBR might deviate more from µFE-based IBR. The continuum stimulus was identified on bone cubes loaded with KUBC, which were also used to identify the homogenized elastic material properties for the hFE models. Other boundary conditions might lead to different exponents in the continuum stimulus as well as to other elastic material properties. The here used target stimulus is a scalar quantity, which ignores other information at the continuum level, such as the orientation of the microstructure. Further, the inclusion of mechanobiological factors, such as metabolism or genetics, in the model might also increase the predictive power of IBR in general. The simplified theory of IBR assumes that the microstructure can fully be explained by mechanical stimuli alone, which holds true only for artificial bone structures.^13^ In general, bone is however influenced by many different factors such as genetics and metabolism.^46^ Recent publications also incorporated mechanoregulation theory^49^ into the target stimulus but required time-lapsed CT to identify remodeling sites in the bone, which is not always available. Only three load cases were applied to the radius sections for simplicity of the models. As these load cases can only predict the reaction force, three rotational load cases should be added for the prediction of moments, to predict physiological loading conditions more accurately.

Despite these limitations, this study could show that µFE-based IBR can be translated to hFE to provide a faster way of predicting physiological loadings from bones. hFE-based IBR was tested on distal radius sections using different kinds of meshing and material mappings. Both µFE and hFE showed a good agreement in terms of predicted load angle if the cortical bone was included in the model and further improved if the bone’s anisotropy is added. The predicted loads correlated well, but systematic differences between µFE and hFE due to the homogenization of the microstructure were observed. Smooth hFE models, including the cortex, showed the best agreement with µFE results. Overall, µFE-based IBR still provides a robust way to infer physiological loading conditions from the bone microstructure, but hFE models offer a computationally more efficient alternative with the ability to model more realistic boundary conditions and more complex load cases.

## Appendix

### Derivation of Optimization Criterion

To use the optimization criterion given in Eq. (2) with finite element models, it has to be discretized first. The SED is measured at the centroid of each of the $k$ element of the FE mesh. The volume of each element ${V}_{j}$ has to be calculated and the density of each element ${\rho }_{j}$ is known from the homogenization. Substituting the integral with a finite sum gives:

6 $${\mathop {{\text{min}}}\limits_{{s_{i} \in \mathbb{R}_{0}^{ + } }}}\; r\left( {s_{i} } \right) = \sum\limits_{{j = 1}}^{k} {\left( {\sum\limits_{{i = 1}}^{n} {\left( {s_{i} U_{{j,i}} } \right)} - \tilde{U}_{0} \rho _{j}^{d} } \right)^{2} } V_{j}$$

This function can be transformed into a matrix form $r({\varvec{x}})=\Vert {\varvec{A}}{\varvec{x}}-{\varvec{b}}\Vert$, in order to be solved efficiently. To write the matrix equations efficiently, ${\varvec{1}}_{m}={\left(1, 1, \ldots ,1\right)}^{T}$ defines a column vector of shape $m\times 1$ filled with ones of arbitrary length. Further, ${\left[{\varvec{A}}\circ {\varvec{B}}\right]}_{ij}={[{\varvec{A}}]}_{ij}[{{\varvec{B}}]}_{ij}$ is the Hadamard product of two matrices of equal size and ${\varvec{a}}\otimes {\varvec{b}}={C}_{ij}={a}_{i}{b}_{j}$ is the dyadic product of two vectors of equal size.

The SED values of all $k$ elements of the FE mesh for the $n$ unit load cases are stored in a matrix ${\varvec{U}}$ of shape $k\times n$, the element volumes in a vector ${\varvec{V}}$ of shape $k\times 1$, and the relative densities for each element in a vector ${\varvec{\rho}}$ of shape $k\times 1$.

Then, the optimization using the scaling vector ${\varvec{s}}$ of shape $n\times 1$, can be written in matrix form as:

7 $${\mathop {{\text{min}}}\limits_{{\varvec{s} \in \{ \mathbb{R}_{0}^{ + } \} ^{n} }}} \;r\left({\varvec{s}}\right)=\Vert {{\varvec{U}}}^{\boldsymbol{\prime}}{\varvec{s}}-\tilde{{\varvec{U}}}\Vert$$

where ${\varvec{U}}^{\boldsymbol{\prime}}$ is the volume corrected matrix of SED, defined as ${{\varvec{U}}}^{\boldsymbol{\prime}}={\varvec{U}}\circ \left(\sqrt{{\varvec{V}}}\otimes {\varvec{1}}_{n}\right)$ and $\tilde{{\varvec{U}}}$ is the target stimulus vector, defined as $\tilde{{\varvec{U}}}={\tilde{U}}_{0}{{\varvec{\rho}}}^{d}\circ \sqrt{{\varvec{V}}}$. The same equation can still be used for optimization of µFE: As ${V}_{j}=\mathrm{const}.$ and ${\rho }_{j}=1$ for each element $j$, the equation will simplify to ${\tilde{{\varvec{U}}}=\tilde{U}}_{0}{\varvec{1}}_{k}$ and ${\varvec{U}}^{\boldsymbol{\prime}}={\varvec{U}}$.

### Homogenized Material Models

The here used material models are either a density dependent power-law model or a density and fabric dependent Zysset-Curnier^51^ type model. While the power-law yields an isotropic model (two independent material parameters), the Zysset-Curnier model is orthotropic (nine independent material parameters).

The power-law uses three parameters: The base elastic-modulus ${E}_{0}$, the base shear modulus ${\mu }_{0}$, and the density exponent $k$. The following equation is used to get the density dependent elastic-modulus:

8 $$E={E}_{0} {\rho }^{k}$$

The Poisson number can be calculated as:

9 $$\nu =\frac{{E}_{0}}{2{\mu }_{0}}-1$$

The Zysset-Curnier model uses, additionally to the density, the eigenvalues ${m}_{i}$ of the fabric tensor, which is a measure for the orientation of the microstructure. The fabric tensor has to be scaled such that the trace of the tensor is equal to 3. An additional exponent $l$ is used to scale the eigenvalues. There are now three elastic-moduli:

10 $${E}_{i}={E}_{0} {\rho }^{k} {m}_{i}^{2l}$$

As well as three shear moduli:

11 $${G}_{ij}={\mu }_{0} {\rho }^{k} {\left({m}_{i}{m}_{j}\right)}^{l}$$

And three independent Poisson numbers:

12 $${\nu }_{ij}={\nu }_{0}{\left(\frac{{m}_{j}}{{m}_{i}}\right)}^{l}$$

In the case of an isotropic material, where all eigenvalues are 1, the model reduces to the power-law model (Eq. (8)), if Eq. (9) is fulfilled for ${\nu }_{0}$.
